# Tunable Backward Terahertz-wave Parametric Oscillation

**DOI:** 10.1038/s41598-018-37068-7

**Published:** 2019-01-24

**Authors:** Kouji Nawata, Yu Tokizane, Yuma Takida, Hiroaki Minamide

**Affiliations:** Tera-Photonics Research Team, RIKEN Center for Advanced Photonics, RIKEN, Sendai, 980-0845 Japan

## Abstract

Backward optical parametric oscillation has attracted attention for cavityless spectral narrowband generation based on perfect photon conversion. Few demonstrations have shown its potential from the aspect of nonlinear photonics; therefore, the mechanisms of momentum conservation among interacting light waves have been concealed by the restricted configuration under the phase-matching condition of periodically poled structures. Here, we unveil a tunable mechanism in the terahertz region by active control of the phase-matching condition. The tunability of backward terahertz-wave parametric oscillation is investigated using a quasi-collinear phase-matching model and its frequency range from the sub-terahertz to terahertz region is identified. Transform-limited terahertz-wave pulse is achieved simply by installing a device on the pump propagating line with no cavity. Moreover, the cascading terahertz-wave generation enhances the photon conversion efficiency, thus making nonlinear optics and its applications more promising. The results highlight new capabilities for using modern ferroelectric materials and encourage further research on nonlinear optics.

## Introduction

Backward optical parametric oscillation (OPO) generates counter-propagating waves to pump waves under a particular wavelength-conversion condition. The theory of backward OPO was originally proposed in 1966^1^; since then, its attractive features such as cavityless narrowband oscillation, compactness, mechanical stability, and perfect photon conversion have been proven through theoretical calculations^2–5^. The investigations have fascinated scientists in nonlinear optics and material science and enabled the realization of promising nonlinear phenomena. In the last decade, the research group at KTH Royal Institute of Technology has reported several demonstrations in the near infrared region by using a periodically poled KTiOPO_4_ (PPKTP) crystal^6–11^. The demonstrations have been realised using the precise technology of crystal fabrication because the large momentum mismatch due to counter-propagating waves must be compensated.

Quasi-phase-matching (QPM) is an indispensable technique for realizing artificial phase-matching-condition through a grating vector to compensate for the momentum mismatch^12,13^. Actually, QPM devices have been used in various applications such as communications^14^, entangled photon generation^15^, and coherent tunable radiation based on OPO^16^. However, backward OPO in terahertz (THz) region has never been demonstrated in practice, even with the QPM technique.

In the THz region, large periodically poled structures of size equal to several tens of microns can compensate the momentum mismatch resulting from the counter-propagating THz-wave. Therefore, backward THz-wave generation based on nonlinear wavelength conversion has been demonstrated by several methods using femtosecond lasers^17–19^, two lasers^20^, or cascaded OPOs^21^. Moreover, alternative wavelength conversion, which is a co-propagating THz-wave generation called forward THz-wave generation, has been observed simultaneously because of the tiny momentum of the THz-wave^17–20^. However, backward THz-wave parametric oscillation (TPO) could not be demonstrated in practice due to the low temporal overlap of femtosecond pulses or high absorption loss of the THz-wave. The practical condition for backward oscillation is rarely found in QPM materials and bulk nonlinear materials.

Generally, vector momentum conservation in QPM can be described as follows: ${{\boldsymbol{k}}}_{{\boldsymbol{pump}}}={{\boldsymbol{k}}}_{{\boldsymbol{signal}}}+{{\boldsymbol{k}}}_{{\boldsymbol{idler}}}+{{\boldsymbol{k}}}_{{\boldsymbol{\Lambda }}}$, where ${{\boldsymbol{k}}}_{{\boldsymbol{pump}}}$, ${{\boldsymbol{k}}}_{{\boldsymbol{signal}}}$, and ${{\boldsymbol{k}}}_{{\boldsymbol{idler}}}$, respectively, denote the wave vectors of the pump, signal, and idler. Further, ${{\boldsymbol{k}}}_{{\boldsymbol{\Lambda }}}$ is the grating vector of the periodically poled QPM structure. In backward OPO, the momentum of the pump vector modified by the grating vector becomes small when compared with that of signal or idler vectors, which is a special and crucial condition to generate the counter-propagating wave. Practically, several demonstrations of backward OPO under collinear phase-matching condition as the simplest interaction configuration have been reported. However, the reduced flexibility of the collinear phase-matching condition provides less tunability of the backward OPO. Therefore, we offer a quasi-collinear phase-matching (QCPM) condition for non-collinear interaction and tunable THz-wave generation, which provides a comprehensive model for backward OPO. Wide tunability in the THz region would provide promising applications such as non-destructive inspection, trace gas sensing, and THz spectroscopy.

## Results

### Tunability of backward THz-wave parametric oscillation

**Figure 1:**
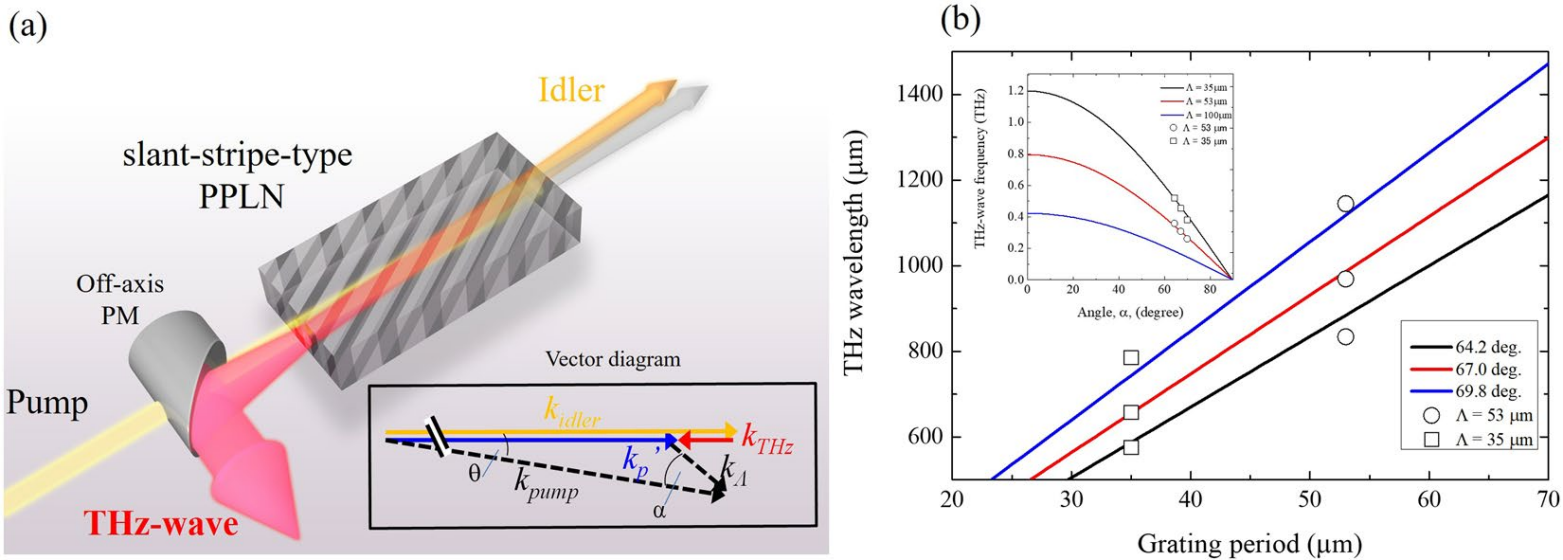
(**a**) Schematic image of backward TPO using a slant-stripe-type PPLN. The grey stripe structure on the PPLN shows the periodically inverted domain. The inset schematic vector diagram shows the phase-matching condition based on QCPM. (**b**) Solid curves and open plots, respectively, show the simulation and experimental results based on QCPM. The open circle and square plots resulted because of using PPLN crystals with poling periods of 53 µm and 35 µm, respectively. The THz wavelength is shown as a function of the grating period with different values of the angle *α* between the pump and grating vectors. The inset graph shows the THz-wave frequency as a function of the angle *α* with different poling periods of PPLN.

Figure 1(a) shows a conceptual diagram of a device for backward TPO with slant-stripe-type periodically poled lithium niobate (PPLN), in which the slanted grating vector is non-parallel to the pump wave. Furthermore, we consider a modified QPM condition based on the following equation: ${{\boldsymbol{k}}}_{{\boldsymbol{p}}}^{{\boldsymbol{\text{'}}}}={{\boldsymbol{k}}}_{{\boldsymbol{THz}}}+{{\boldsymbol{k}}}_{{\boldsymbol{idler}}}=\,{{\boldsymbol{k}}}_{{\boldsymbol{pump}}}-{{\boldsymbol{k}}}_{{\boldsymbol{\Lambda }}}$. The momentum vector diagram is shown within the inset in Fig. 1(a), in which the magnitudes of the vectors satisfy the relational expression of $|{{\boldsymbol{k}}}_{{\boldsymbol{idler}}}| > |{{\boldsymbol{k}}}_{{\boldsymbol{p}}}^{{\boldsymbol{\text{'}}}}| > |{{\boldsymbol{k}}}_{{\boldsymbol{THz}}}|$ in the backward configuration. By introducing ${{\boldsymbol{k}}}_{{\boldsymbol{p}}}^{{\boldsymbol{\text{'}}}}$, the phase-matching condition harvests a new collinear-interaction scheme of ${{\boldsymbol{k}}}_{{\boldsymbol{p}}}^{{\boldsymbol{\text{'}}}}\parallel {{\boldsymbol{k}}}_{{\boldsymbol{THz}}}\parallel {{\boldsymbol{k}}}_{{\boldsymbol{idler}}}$, defined as the quasi-collinear phase-matching (QCPM) condition. In reality, the scheme is realised in a small angle *θ* between the vectors of ${{\boldsymbol{k}}}_{{\boldsymbol{pump}}}$ and ${{\boldsymbol{k}}}_{{\boldsymbol{p}}}^{{\boldsymbol{\text{'}}}}$ because of the interaction volume of the pump, idler, and THz-waves.

In the experiment, a slant-stripe-type PPLN with crystal length of 50 mm, poling period of 53 μm or 35 μm, and slanted angle of 67° was used. The input and output faces were coated for antireflection at the pump and idler wavelengths. This coating had no effect on the THz-wave output because the coating thickness was less than the THz wavelength. A single-longitudinal-mode sub-nanosecond Nd:YAG laser was used as a master oscillator for pumping the crystal. The pulse width and wavelength were 660 ps and 1064.34 nm, respectively. The wavelength linewidth was 0.01 nm measured by an optical spectrum analyser with spectrum resolution of 0.01 nm. The corresponding frequency bandwidth was limited to 3 GHz due to the resolution. The pulse energy of the pump beam was amplified up to 8.9 mJ by a master oscillator power amplifier consisting of a diode pumped Nd:YAG amplifier with a four-pass configuration. The pump beam incident on the PPLN was collimated to 0.78-mm diameter (full width at half maximum; FWHM) by a lens pair. Polarisation of the pump beam was aligned along the z-axis of the PPLN crystal by a half-wave plate. For separation of the backward THz wave from the pump beam, an off-axis parabolic mirror (PM) with a hole for the pump beam was used with different beam divergence between the two. In contrast, the generated idler propagating forward was spatially separated from the pump beam under the QCPM condition. The idler wave and the THz wave were measured, respectively, with an optical power-meter and a 4 K Si-bolometer.

The simulation results based on the model described above are presented in Fig. 1(b) and its inset. The tunable THz-wave wavelength and frequency are shown, respectively, as functions of the grating period and the angle *α*. The solid lines were calculated at THz-wave wavelengths with angle *α* of values 64.2°, 67.0°, and 69.8°. The pump wavelength was fixed at 1064.34 nm in the calculation, which corresponded to the fundamental emission of an Nd:YAG laser. The inset shows a tunable range of up to 1.2 THz using a PPLN with grating period of 35 μm. A shorter grating period is necessary to obtain frequencies higher than 1.2 THz. However, a crystal with a grating period less than 35 μm innately satisfies the phase-matching condition for conventional OPG^22^. The undesirable effects of OPG might interrupt the parametric process of a backward TPO.

Tunability of the backward TPO can be achieved by practical control of the angle *α* between the pump and grating vectors by rotating the nonlinear crystal. The experimental results are plotted in Fig. 1(b) as open circles and squares. The idler wavelengths were also measured using the optical spectrum analyser. The idler wavelengths of 1065.33 nm, 1065.51 nm, and 1065.70 nm were measured using the PPLN with a poling period of 53 μm at angles of 64.2°, 67.0°, and 69.8°, respectively. The corresponding THz-wave wavelengths were 1145 μm, 969 μm, and 834 μm, respectively. The tuning range of approximately 100 GHz was restricted by an angular aperture of 5.6° from the crystal centre. Because the simulation shows the tunability to be wider, another PPLN was designed and investigated. This PPLN had a poling period of 35 μm and a slanting angle of 67°. The obtained wavelengths of the idler were 1065.79 nm, 1066.10 nm, and 1066.31 nm when the angle between the pump and the grating vector was set at 64.2°, 67.0°, and 69.8°, respectively. The corresponding THz-wave frequencies agreed well with the simulation curve, as presented in Fig. 1(b). These results indicate that rapid frequency tunability by rotating the crystal provides high-speed THz-wave inspection, sensing, and spectroscopy. A tiny non-collinear angle *θ* within 1° would allow full tunability by using a disk-shaped crystal in practice.

### Narrowband oscillation

**Figure 2:**
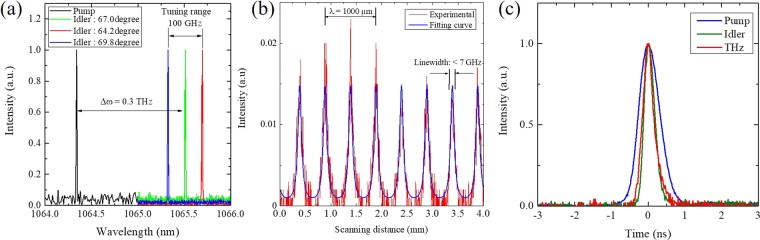
(**a**) Measured spectra of pump and idler waves. Blue, green, and red lines show the spectra for the angle *α* of 64.2°, 67.0°, and 69.8°, respectively. (**b**) Measured THz-wave intensity through the etalon cavity as a function of scanning distance at angle *α* of 67.0°. Red and blue solid lines show the experimental and numerical results, respectively. The PPLN with grating period of 53 μm was used for both measurements. (**c**) Measured pulse widths of the pump, idler, and THz-wave.

The spectrum bandwidth is another proof of the backward TPO because of cavityless configuration. Narrowband oscillation is an inherent characteristic of backward-wave OPO, which can be explained by phase-matching condition between counter-propagating photons. Counter-propagating parametric photons, where the signal and idler waves propagate in the opposite directions, enforces strong constraints on the momentum mismatch. Thus, severe constraint on the spectrum provides narrowband oscillation. Figure 2(a) presents the spectra of the pump and idlers given by the optical spectrum analyser at a spectral resolution of 0.01 nm. Generally, the spectrum width of the generated idler is wide in a typical OPG process because of a broadband gain, even though a narrow-band pump beam is used. However, in backward oscillation, a narrow bandwidth comparable to that of the pump would be generated because of the inherent feedback effect. In the experiment, the narrow-band pump spectrum below 0.01 nm was irradiated into the PPLN and similar bandwidths of the idlers were measured as presented in Fig. 2(a).

Figure 2(b) also shows that a narrow-band oscillation was obtained. Measurements of the THz-wave wavelength and oscillation bandwidth were performed using a scanning Fabry–Pérot etalon consisting of two parallel silicon plates acting as partial reflectors. The variation in the displacement of one silicon plate produced a clear interference pattern with peaks, which was fitted into the calculation curve. The result shows a wavelength of approximately 1000 μm (0.3 THz in frequency) and a narrow bandwidth of less than 7 GHz at a free spectral range of 37 GHz, with finesse of 6 for the etalon. Consequently, the inherent feed-back effect in the backward TPO achieved the near Fourier-transform-limited bandwidth using no spectrum-narrowing technique such as a cavity for resonance or injection seeding.

The temporal pulse widths also indicate narrowband oscillation. Figure 2(c) shows the temporal pulse widths for the pump, idler, and THz-wave. They were measured by using a photodetector for optical pulses and a Schottky barrier diode for THz-wave. The pulse widths of the THz-wave and the idler became half of the pump width through the nonlinear process. The pulse widths were approximately 340 ps at FWHM. The corresponding frequency bandwidths from the pulse widths were approximately 1.3 GHz, which was almost equivalent to the results of the scanning etalon. The backward TPO can allow the generation of near Fourier transform-limited pulses because of the strong feedback effect of the counter-propagating waves.

### Backward, forward, and cascading processes

**Figure 3:**
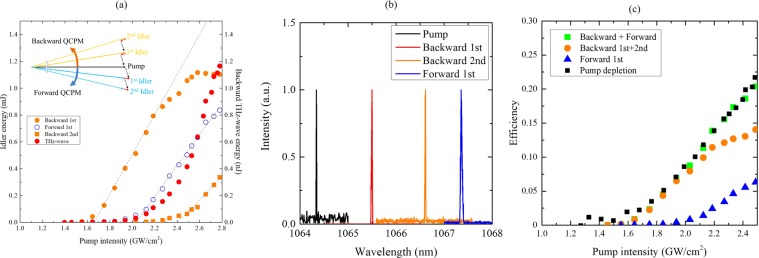
(**a**) Idler and THz-wave energies as functions of pump intensity. The inset shows QCPM conditions for both backward and forward configurations, including the cascading processes. (**b**) Pump and idler spectra for backward TPO and forward TPG. (**c**) Energy conversion efficiency from pump to idler waves. Pump depletion rate is plotted as black squares.

Figure 3(a) shows the idler and THz-wave energy as a function of pump intensity, including results based on both the backward and forward QCPM conditions. The inset figure shows the vector diagram. The first idler and THz-wave attributed to the backward TPO was obtained at pump intensities higher than 1.6 GW/cm^2^. The idler energy increased linearly with a slope of 1.4 mJ/(GW∙cm^−2^), reaching 1.1 mJ with a pump intensity of approximately 2.6 GW/cm^2^. Above an original pump intensity of 2.4 GW/cm^2^, a second idler wave with wavelength of 1066.61 nm was obtained with a slope of 0.83 mJ/(GW∙cm^−2^), which was based on the cascading process of the backward TPO. The output THz-wave energy was nonlinearly increased when compared with the idler energy due to the cascade process and the absorption loss inside the crystal. The output energy of 1.2 nJ was achieved at the maximum pump intensity and no saturation was observed in this experiment. Another idler wave of the forward QCPM TPG was also achieved above a pump intensity of 2.0 GW/cm^2^. The forward idler wavelength of 1067.36 nm was completely separated from those of the backward TPOs. The idler wavelength of the forward TPG also matched with that calculated for the forward QCPM condition.

In comparison with the backward phase matched idlers, the spectrum of the forward phase matched idler was another proof of backward wave oscillation. Figure 3(b) shows the pump and idler wavelengths of the backward TPO and forward TPG processes. The idler wavelengths were completely separated from each other. The frequency differences between the pump and idler waves well matched the calculated result based on the QCPM condition. The bandwidth of the backward processes was 0.01 nm, which was equivalent to that of a near Fourier transform-limited pulse. On the other hand, the bandwidth of forward TPG was relatively broad at approximately 0.03 nm. The feedback effect provides monochromatic THz-wave oscillation without any cavity configuration.

According to the calculation of the phase-matching condition, THz-wave frequencies of the backward process and forward process were 0.3 THz and 0.8 THz, respectively. The corresponding absorption coefficient of LN was approximately 1.8 cm^−1^ at 0.3 THz and 7.6 cm^−1^ at 0.8 THz from our measurement using THz time-domain spectroscopy. Therefore, the backward TPO process had the highest parametric gain because of relatively low absorption loss for THz-wave compared with that in the forward THz-wave generation process. The threshold pump-intensity of the backward TPO was nearly 20% below that of the forward TPG. Furthermore, the efficient energy conversion faced the second threshold to the cascading backward TPO process, in which the power intensity of the first idler wave was higher than 1.6 GW/cm^2^. The result indicates that the cascading process will allow us to achieve more efficient wavelength-conversion over the Manley–Rowe limit.

To confirm the cascading process, the THz frequency should be compared with the calculated frequency based on the phase matching condition mentioned above. The calculated backward THz frequency became relatively low through the cascading process. The THz frequency difference between the 1^st^ and 2^nd^ processes was approximately 6 GHz. In practice, the frequency difference was approximately 10 GHz. These results well explained the physical behaviour of cascaded backward oscillation. The frequency shift in this experiment mainly originates from the angular shift between the pump vector and the 1^st^ idler vector, as shown in the inset of Fig. 3(a). Another PPLN satisfying the collinear phase-matching condition can provide monochromatic THz-wave generation with cascaded backward oscillation.

Figure 3(c) shows the energy conversion efficiency from pump to idler waves and the pump depletion. The photon energy of the THz-wave was 3 orders of magnitude smaller than that of the idler wave. Therefore, pump depletion can be discussed in terms of the energy conversion efficiency from the pump to the idler waves. The conversion efficiency of the backward process increased linearly with the pump intensity of up to 2.2 GW/cm^2^. The kink at the intensity of 2.2 GW/cm^2^ was caused by the forward TPG process. Nevertheless, the conversion efficiency was not saturated and reached 0.15. In general, the efficiency of the backward OPOs does not exhibit saturation or roll-off caused by back-conversion to the pump, which typically occurs in conventional OPOs with co-propagating interactions. The strong suppression of back-conversion and absence of other competing parasitic processes except for the forward TPG allow further rise in pump intensity, thereby reaching high efficiencies. Efficient backward TPO can be expected with high pump intensity up to the optical damage threshold, which was approximately 5 GW/cm^2^ for MgO doped LN crystals.

In comparison to an injection-seeded THz-wave parametric generation (is-TPG) using bulk LN, backward TPO has the significant advantage of a simple scheme combining a microchip laser as a pump source and a nonlinear crystal. The photon conversion efficiency was also equivalent to that of the is-TPG system because of the inhibit feedback effect. The advantage makes backward TPO a suitable field deployable source for highly demanding real-world chemical sensing applications. Moreover, THz-wave parametric generation in the sub-THz region using bulk LN would be difficult because of the low parametric gain. For example, the parametric gain at 0.3 THz was almost a quarter of that at 2 THz, which was the frequency with the maximum parametric gain in bulk LN^23^. Backward TPO is a promising technique that extends photonics technology to the sub-THz frequency region.

## Discussion

We compared our results with those of the conventional backward THz-wave generation method based on DFG. The observation of backward THz-wave frequency of 300 GHz pumped by the narrowband pump source with the maximum frequency bandwidth of 3 GHz demonstrates backward TPO occurring in the PPLN crystal. The significant threshold intensity and the non-collinear phase-matching condition were completely different behaviours compared with those of backward THz-wave generation based on the DFG process, which should satisfy the collinear phase-matching condition^18^. Based on the above facts, our work presents a completely different physical process from the conventional backward THz radiation. Moreover, backward TPO as a novel physical phenomenon was achieved.

The frequency tunability supported by theoretical calculation and experimental results is wide enough to consider applications such as non-destructive inspection, trace gas sensing, and THz spectroscopy. Backward TPO also shows excellent potential for compact and high conversion efficiency. These advantages make backward TPO a suitable field deployable source for most demanding real-world chemical sensing applications. Backward TPO provides a simple scheme compared with the conventional bulky THz system and will bring a paradigm shift in the field of THz technology.

In conclusion, this report describes a study of QCPM in non-collinear interaction, with demonstration of a tunable backward TPO using a slant-stripe-type PPLN. The engineered momentum vector provides a sophisticated phase-matching condition for the pump and grating vectors. The mechanism of the tunable backward TPO is clarified by the concept of QCPM. The concept also encourages the study of the complicated phase-matching condition of PPLN in other wavelength regions. Additionally, the property of complete photon conversion in backward OPO suggests that higher conversion efficiency can be achieved in backward TPO through further study of a cascading process that breaks the Manley–Rowe limit. Furthermore, the technique of backward TPO is expected to be applicable in efficient THz-wave parametric amplification^24^ and sensitive THz-wave detection with nonlinear up-conversion detection^25^. This simple device further promotes THz-wave research and applications because of the mechanical stability, compactness, and robustness, which are achieved simply by installing the device on the pump propagating line.

## Methods

### Pump pulse width and efficient THz-wave generation

A single-longitudinal-mode microchip Nd:YAG laser was used as a master oscillator for the pump source. The pulse width of 660 ps was suitable for efficient THz-wave generation because it avoided competition between stimulated polariton scattering and stimulated Brillouin scattering^26^. Additionally, the pulse width corresponded to the spatial length of 20 cm, which was much longer than the PPLN crystal. Strong interactions in the temporal and spatial regions between counter-propagating waves inside the crystal provided effective wavelength conversion.

## References

[1] Harris SE (1966). Proposed backward wave oscillation in the infrared. Appl. Phys. Lett..

[2] Hsu H, Yu C (1973). Complete photon conversion in backward-travelling-wave parametric amplification and oscillation. Electro. Lett..

[3] Ding YJ, Khurgin JB (1996). Mirrorless optical parametric oscillators. J. Nonlinear Opt. Phys. Mater..

[4] Ding YJ, Khurgin JB (1998). A new scheme for efficient generation of coherent and incoherent submillimeter to THz waves in periodically-poled lithium niobate. Opt. Commun..

[5] Chuu C-S, Harris SE (2011). Ultrabright backward-wave biphoton source. Phys. Rev. A.

[6] Canalias C, Pasiskevicius V (2007). Mirrorless optical parametric oscillator. Nat. Photonics.

[7] Strömqvist G (2012). Temporal coherence in mirrorless optical parametric oscillators. J. Opt. Soc. Am. B.

[8] Pasiskevicius V, Strömqvist G, Laurell F, Canalias C (2012). Quasi-phase matched nonlinear media: progress towards nonlinear optical engineering. Opti. Mater..

[9] Strömqvist G, Pasiskevicius V, Canalias C (2011). Self-established noncollinear oscillation and angular tuning in a quasi-phase-matched mirrorless optical parametric oscillator. Appl. Phys. Lett..

[10] Zukauskas A, Viotti AL, Liljestrand C, Pasiskevicius V, Canalias C (2017). Cascaded counter-propagating nonlinear interactions in highly-efficient sub-μm periodically poled crystals. Sci. Rep..

[11] Liljestrand C, Zukauskas A, Pasiskevicius V, Canalias C (2017). Highly efficient mirrorless optical parametric oscillator pumped by nanosecond pulses. Opt. Lett..

[12] Armstrong JA, Bloembergen N, Ducuing J, Pershan PS (1962). Interactions between light waves in a nonlinear dielectric. Phys. Rev..

[13] Fejer MM, Magel GA, Jundt DH, Byer RL (1992). Quasi-phase-matched second harmonic generation: tuning and tolerances. IEEE. J. Quantum Electron..

[14] Chou MH, Hauden J, Arbore MA, Fejer MM (1998). 1.5-µm-band wavelength conversion based on difference-frequency generation in LiNbO_3_ waveguides with integrated coupling structures. Opt. Lett..

[15] Tanzilli S (2002). PPLN waveguide for quantum communication. Eur. Phys. J. D.

[16] Myers LE, Eckardt RC, Fejer MM, Byer RL, Bosenberg WR (1996). Multigrating quasi-phase-matched optical parametric oscillator in periodically poled LiNbO_3_. Opt. Lett..

[17] Lee Y-S (2000). Generation of narrow-band terahertz radiation via optical rectification of femtosecond pulses in periodically poled lithium niobate. Appl. Phys. Lett..

[18] Weiss C (2001). Tuning characteristics of narrowband THz radiation generated via optical rectification in periodically poled lithium niobate. Opt. Express.

[19] Yu NE (2008). Simultaneous forward and backward terahertz generations in periodically poled stoichiometric LiTaO_3_ crystal using femtosecond pulses. Appl. Phys. Lett..

[20] Wang TD, Lin ST, Lin YY, Chiang AC, Huang YC (2008). Forward and backward terahertz-wave difference-frequency generations from periodically poled lithium niobate. Opt. Express..

[21] Sowade R (2009). Continuous-wave optical parametric terahertz source. Opt. Express.

[22] Umemura N, Matsuda D, Mizuno T, Kato K (2014). Sellmeier and thermo-optic dispersion formulas for the extraordinary ray of 5 mol. % MgO-doped congruent LiNbO_3_ in the visible, infrared, and terahertz regions. Appl. Opt..

[23] Hayashi S (2016). High-Brightness Continuously Tunable Narrowband Subterahertz Wave Generation. IEEE Trans. Terahertz Sci. Technol..

[24] Taira Y (2014). A terahertz wave parametric amplifier with a gain of 55 dB. IEEE Trans. Terahertz Sci. Technol..

[25] Nawata K (2014). Effective terahertz-to-near-infrared photon conversion in slant-stripe-type periodically poled LiNbO_3_. Appl. Phys. Lett..

[26] Nawata K (2017). Effective Terahertz Wave Parametric Generation Depending on the Pump Pulse Width Using a LiNbO_3_ Crystal. IEEE Trans. Terahertz Sci. Technol..

